# Maximizing *T*_c_ by tuning nematicity and magnetism in FeSe_1−*x*_S_*x*_ superconductors

**DOI:** 10.1038/s41467-017-01277-x

**Published:** 2017-10-26

**Authors:** K. Matsuura, Y. Mizukami, Y. Arai, Y. Sugimura, N. Maejima, A. Machida, T. Watanuki, T. Fukuda, T. Yajima, Z. Hiroi, K. Y. Yip, Y. C. Chan, Q. Niu, S. Hosoi, K. Ishida, K. Mukasa, S. Kasahara, J.-G. Cheng, S. K. Goh, Y. Matsuda, Y. Uwatoko, T. Shibauchi

**Affiliations:** 1 Department of Advanced Materials Science, University of Tokyo, Kashiwa, Chiba 277-8561 Japan; 2 Synchrotron Radiation Research Center, National Institutes for Quantum and Radiological Science and Technology, Sayo, Hyogo 679-5148 Japan; 3 Materials Sciences Research Center, Japan Atomic Energy Agency (SPring-8/JAEA), Sayo, Hyogo 679-5148 Japan; 4 Institute for Solid State Physics, The University of Tokyo, Kashiwa, Chiba 277-8581 Japan; 5 Department of Physics, The Chinese University of Hong Kong, Shatin, Hong Kong; 6 Department of Physics, Kyoto University, Sakyo-ku, Kyoto 606-8502 Japan; 7 Beijing National Laboratory for Condensed Matter Physics and Institute of Physics, Chinese Academy of Sciences, 100190 Beijing, China

## Abstract

A fundamental issue concerning iron-based superconductivity is the roles of electronic nematicity and magnetism in realising high transition temperature (*T*_c_). To address this issue, FeSe is a key material, as it exhibits a unique pressure phase diagram involving non-magnetic nematic and pressure-induced antiferromagnetic ordered phases. However, as these two phases in FeSe have considerable overlap, how each order affects superconductivity remains perplexing. Here we construct the three-dimensional electronic phase diagram, temperature (*T*) against pressure (*P*) and isovalent S-substitution (*x*), for FeSe_1−*x*_S_*x*_. By simultaneously tuning chemical and physical pressures, against which the chalcogen height shows a contrasting variation, we achieve a complete separation of nematic and antiferromagnetic phases. In between, an extended non-magnetic tetragonal phase emerges, where *T*_c_ shows a striking enhancement. The completed phase diagram uncovers that high-*T*_c_ superconductivity lies near both ends of the dome-shaped antiferromagnetic phase, whereas *T*_c_ remains low near the nematic critical point.

## Abstract (web summary)

The overlap between different phases has hindered the understanding of how each phase affects superconductivity in FeSe. Here, Matsuura et al. achieve a complete separation of non-magnetic nematic and antiferromagnetic phases for FeSe_1-*x*_S_*x*_, observing a tetragonal phase in between with a strikingly enhanced *T*_c_.

## Introduction

One of the common aspects among unconventional superconductors, including high-*T*_c_ cuprates, heavy-fermion and organic materials, is the appearance of a superconducting dome in the vicinity of magnetic order. This has naturally led to the notion of superconducting pairing mechanism driven by magnetic fluctuations^1,2^. In iron pnictides, high-*T*_c_ superconductivity also appears near the antiferromagnetic phase^3^, which however is accompanied by the tetragonal-to-orthorhombic structural transition with significant electronic anisotropy (nematicity). This gives rise to new theoretical proposals involving the fluctuations of this electronic nematicity as a glue for the electron pairing^4–6^. Although enhanced nematic fluctuations of ferro-type (*q* = 0) are observed experimentally^7^, the antiferromagnetic fluctuations are also enhanced^8^, and thus it is difficult to pinpoint the impact of nematic fluctuations on the superconductivity in iron pnictides.

From this viewpoint, the FeSe-based superconductors are a suitable system for addressing the importance of nematic fluctuations, as it has a unique phase diagram^9^. At ambient pressure, FeSe shows a nematic transition at *T*_s_ = 90 K without magnetic order down to the lowest temperature^10^. Under pressure, antiferromagnetic order is induced^11–16^, and the superconducting *T*_c_ is enhanced by more than a factor of 4^9,17^. Recently, it has been shown that the nematic transition can be tuned to a quantum critical point by isovalent substitution of Se with S, but without inducing magnetic order^18^. These results indicate the non-equivalence of physical and chemical pressure in this system. This implies that one can control the magnetism and nematicity independently by these tuning knobs, isovalent substitution and physical pressure, which offers the possibility to disentangle intertwined effects of nematic and magnetic fluctuations on high-*T*_c_ superconductivity.

Here we present our systematic study of temperature-pressure-substitution (*T*-*P*-*x*) phase diagrams of FeSe_1−*x*_S_*x*_ in wide ranges of pressure (up to *P* ~ 8 GPa) and sulphur content (0 ≤ *x* ≤ 0.17). In pure FeSe, it has been shown by several groups that the nematic transition temperature *T*_s_ is suppressed by pressure (*P* < 2 GPa) but before the complete suppression of *T*_s_, antiferromagnetic or spin density wave (SDW) order is induced, resulting in an overlap region of these two phases^9,12–15^. With increasing *x*, the nematic transition temperature *T*_s_ is lowered and correspondingly the nematic phase is rapidly suppressed by pressure. However, an opposite trend is found for pressure-induced magnetism: the SDW onset pressure is shifted to higher pressure. These lead to the emergence of the tetragonal non-magnetic phase in between, which becomes wider with increasing *x*. Most importantly, a new high-*T*_c_ superconducting dome emerges in the tetragonal phase. Based on the obtained three-dimensional phase diagram, we discuss the relationship between the two orders and superconductivity in this system.

## Results

### Phase diagram

**Fig. 1:**
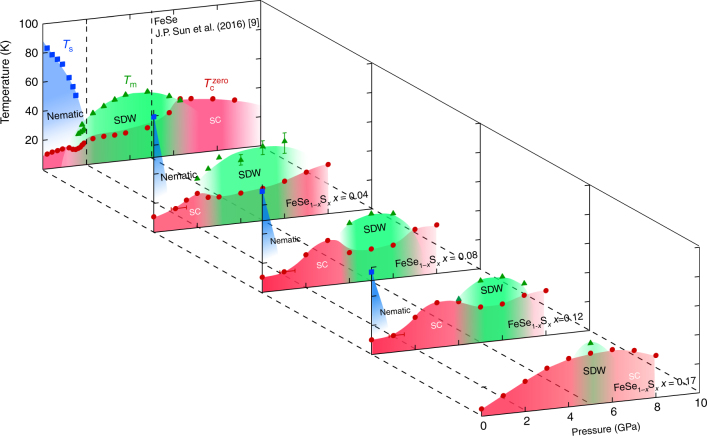
Temperature-pressure-concentration phase diagram in FeSe_1−*x*_S_*x*_. The structural (*T*_s_, blue squares), magnetic (*T*_m_, green triangles) and superconducting transition temperatures ($T_{\rm{c}}^{{\rm{zero}}}$, red circles) are plotted against hydrostatic pressure *P* and *S* content *x*. Following the procedure reported for *x* = 0 by Sun et al.^9^, *T*_s_, *T*_m_ and $T_{\rm{c}}^{{\rm{zero}}}$ are defined respectively by the temperatures of upturn, kink and zero resistivity in *ρ*(*T*) curves measured in the constant-loading type cubic anvil cell for *x* = 0.04, 0.08, 0.12 and 0.17 (Supplementary Figs. 3–6). The errors of *T*_m_, are estimated from the broadness of the kink anomaly in *ρ*(*T*). The cell is optimized for the high-pressure range, and thus for *P* < 2 GPa the error of pressure is relatively large (see error bars for 1 GPa) compared to higher pressures. The colour shades are the guides for the eyes. Detailed phase diagrams for constant *x* and *P* are shown in Supplementary Figs. 1, 2, respectively

**Fig. 2:**
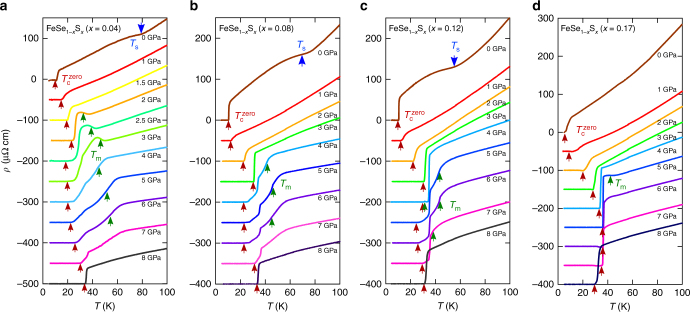
Evolution of temperature-dependent resistivity under pressure in FeSe_1−*x*_S_*x*_. **a**–**d** *ρ*(*T*) curves below 100 K at different pressures up to 8.0 GPa measured for *x* = 0.04 (**a**), 0.08 (**b**), 0.12 (**c**) and 0.17 (**d**). The data are vertically shifted for clarity. The resistive anomalies at transition temperatures *T*_s_ (blue), *T*_m_ (green) and $T_{\rm{c}}^{{\rm{zero}}}$ (red) are indicated by the arrows. For *x* = 0.04 (**a**), the anomalies associated with the magnetic transition is smeared and thus the error of *T*_m_ determination is relatively large for *P* ≥ 4 GPa (see error bars in Fig. 1)

In FeSe_1−*x*_S_*x*_ at ambient pressure, the temperature dependence of resistivity *ρ*(*T*) for *x* < 0.17 exhibits a slight upturn upon cooling at *T*_s_ due to tetragonal-to-orthorhombic structural transitions, and then it goes to zero below the superconducting transition temperature $\left( {T_{\rm{c}}^{{\rm{zero}}}} \right)$ ^18^. By measuring *ρ*(*T*), we determine the structural transition temperature (*T*_s_) and $\left( {T_{\rm{c}}^{{\rm{zero}}}} \right)$ for *x* = 0.04, 0.08 and 0.12 at ambient pressure as shown in the electronic phase diagram for different S contents as shown in Fig. 1 (see also Supplementary Note 1, Supplementary Figs. 1, 2 for two-dimensional slices). For *x* = 0.17, we do not observe any signature of the structural transition, indicating the complete suppression of *T*_s_ as reported previously^18^. In Fig. 2a–d, we show the evolution of *ρ*(*T*) under pressure measured using a cubic anvil cell (CAC) which can generate pressure with a good hydrostatic condition and maintain constant pressure upon cooling^19^. With applying pressure, the *T*_s_ anomaly observed at ambient pressure in *x* = 0.04, 0.08 and 0.12 disappears completely at *P* ≲ 1 GPa. This is a natural consequence of the fact that both S substitution and applying pressure suppress the structural transition in FeSe.

In *x* = 0.04, the *ρ*(*T*) curve at 2.0 GPa exhibits a clear upturn around 40 K. The temperature of the upturn increases with pressure, and then it turns to a kink above 4.0 GPa. This evolution of resistive transition is reminiscent of the magnetic transition seen in FeSe under pressure^9^. Therefore, we follow the procedure of ref. ^9^ to determine the magnetic transition temperatures (*T*_m_) by using a dip or peak in d*ρ*/d*T* (Supplementary Note 2 and Supplementary Figs. 3–6), and the pressure-evolution of *T*_m_ is shown in Fig. 1 and Supplementary Fig. 1b. With increasing pressure, *T*_m_ is enhanced monotonically up to 6.0 GPa, while $T_{\rm{c}}^{{\rm{zero}}}$ determined by the zero resistivity is slightly suppressed just after the emergence of magnetism. Inside the magnetic phase, the superconducting transition in *ρ*(*T*) becomes broad. When we define $T_{\rm{c}}^{{\rm{peak}}}$ as the peak temperature in d*ρ*(*T*)/d*T* (Supplementary Fig. 3), the difference between $T_{\rm{c}}^{{\rm{zero}}}$ and $T_{\rm{c}}^{{\rm{peak}}}$ is significant in the magnetic phase (Supplementary Fig. 1). Above 7.0 GPa, the kink anomaly due to the magnetic transition disappears, and concomitantly $T_{\rm{c}}^{{\rm{zero}}}$ increases gradually up to 32 K, resembling the evolution of the electronic phases in FeSe at high pressure^9^.

**Fig. 3:**
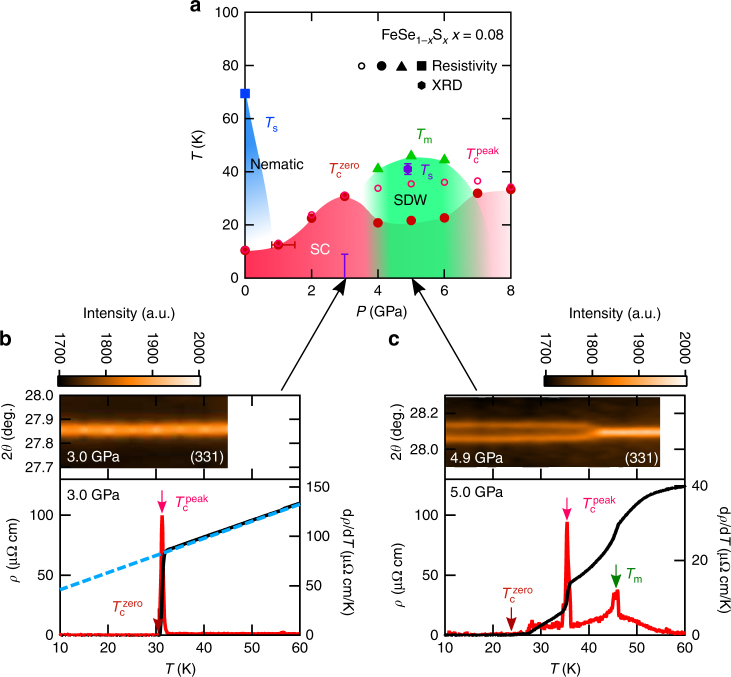
Temperature-pressure phase diagram for *x* = 0.08. **a** *T*−*P* phase diagram of FeSe_1−*x*_S_*x*_ (*x* = 0.08) together with *T*_s_ determined by the high-pressure synchrotron X-ray diffraction (XRD) in a diamond anvil cell (purple hexagon with error bars). **b**, **c** Temperature dependence of Bragg intensity as a function of 2*θ* angle is indicated in colour scale for 3.0 GPa (**b**) and 4.9 GPa (**c**). *ρ*(*T*) and d*ρ*/d*T* are also shown with the same horizontal axis. The red, pink and green arrows indicate $T_{\rm{c}}^{{\rm{zero}}}$, $T_{\rm{c}}^{{\rm{peak}}}$ and *T*_m_, respectively. The blue dashed line in **b** is a *T*-linear fit to the normal-state *ρ*(*T*) at 3.0 GPa

In *x* = 0.08 and 0.12, we observe remarkable features at moderate pressures. As shown in Fig. 2b, c, there is no discernible upturn anomaly in *ρ*(*T*) between 1.0 and 3.0 GPa for *x* = 0.08 and 0.12. At 3.0 GPa, a clear *T*-linear behaviour in the normal-state resistivity is observed (Fig. 3b), which is accompanied by a sharp superconducting transition with enhanced $T_{\rm{c}}^{{\rm{zero}}}$ of ~32 K. We checked for *x* = 0.12 that *T*_c_ determined by ac susceptibility is consistent with that determined by the zero resistivity (Supplementary Note 3, Supplementary Figs. 7, 8). Further increase of pressure leads to the emergence of magnetism seen as the kink anomaly around 40 K, then it persists up to 6.0 (7.0) GPa for *x* = 0.08 (0.12). The marked difference compared with FeSe under pressure is the strong enhancement of *T*_c_ in the lower pressure side of the magnetic phase, forming a peak in $T_{\rm{c}}^{{\rm{zero}}}$ around 3.0 GPa for both *x* = 0.08 and 0.12. With increasing pressure above 7.0 (8.0) GPa in *x* = 0.08 (0.12), the kink anomaly due to the magnetic transition disappears and $T_{\rm{c}}^{{\rm{zero}}}$ exhibits another gradual enhancement up to ~32 K after the disappearance of *T*_m_, resulting in the double-dome structure in $T_{\rm{c}}^{{\rm{zero}}}$ having two maxima with almost identical magnitudes.

In *x* = 0.17, where there is no *T*_s_ at ambient pressure as shown in Fig. 2d, its initial $T_{\rm{c}}^{{\rm{zero}}}$ of ~4 K gradually increases up to ~35 K with pressure, and turns to decrease above 6.0 GPa, forming a broad superconducting dome as a function of pressure as illustrated in Fig. 1. We observe *T*_m_ only at 5.0 GPa for this S content, implying that the system is approaching the verge of the pressure-induced SDW phase (Supplementary Fig. 2f).

### Emergent tetragonal phase

As *x* is increased, the pressure-induced SDW dome shifts to higher pressure and shrinks, while low-pressure non-magnetic nematic phase shifts to lower pressure and disappears at *x* ~ 0.17. We stress that the nematic phase is completely separated from the SDW phase at *x* ≥ 0.04. To confirm the separation between two distinct phases under pressure, we performed synchrotron X-ray diffraction measurements under pressure for *x* = 0.08 (Fig. 3a, and Supplementary Note 4). In Fig. 3b, c, we show (331) Bragg intensity as a function of temperature at 3.0 and 4.9 GPa together with the *ρ*(*T*) and d*ρ*/d*T* data. At 3.0 GPa, no discernible change of the Bragg-peak is observed down to the lowest temperature of 10 K (Fig. 3b and Supplementary Fig. 9a). At 4.9 GPa, on the other hand, the splitting of the Bragg peak is clearly resolved around ${T_{\rm{s}}}\sim41$ K, evidencing the presence of the tetragonal-to-orthorhombic structural transition (Fig. 3c and Supplementary Fig. 9b). This structural transition is located very close to the SDW transition at *T*_m_ at 5.0 GPa as indicated by the sharp peak in d*ρ*/d*T* curve in Fig. 3b. Thus it is natural to consider that the magnetic phase has an orthorhombic structure, similar to the case of the high-pressure SDW phase of FeSe^13,14^.

These results demonstrate that the high-*T*_c_ superconductivity in FeSe_1−*x*_S_*x*_ is realised in the tetragonal phase newly emerged between the orthorhombic nematic and magnetic phases. In the non-magnetic tetragonal phase (1 GPa ≲ *P* ≲ 3 GPa), *T*_c_ shows a strong increase with *P* (Figs. 1, 3a), indicating that the enhancement of superconductivity is most pronounced near the verge of the magnetic phase, not the nematic phase. It is also likely that the *T*-linear resistivity observed near the SDW boundary (Fig. 3b) is a consequence of enhanced antiferromagnetic fluctuations, as reported in other high-*T*_c_ cases^8,20,21^.

## Discussion

**Fig. 4:**
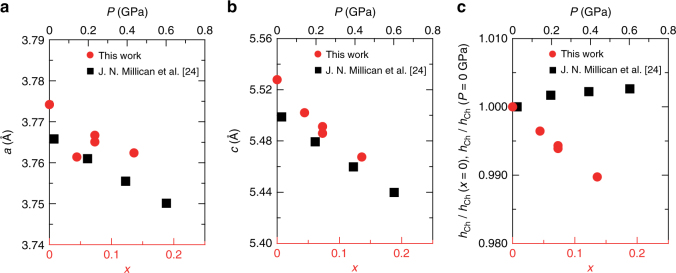
Comparisons between physical pressure and isovalent substitution effects on the structural parameters. **a**, **b** Lattice constants *a* (**a**) and *c* (**b**) as a function of S content *x* in the present single crystals of FeSe_1−*x*_S_*x*_ (red circles, bottom axis), compared with those as a function of pressure reported for polycrystals of FeSe in ref. ^24^ (black squares, top axis). **c** Chalcogen height *h*_Ch_ normalized by the initial values as a function of *x* (red circles, bottom axis) and pressure (black squares, top axis)^24^. The numerical values of these parameters are listed in Supplementary Table 1

Why the effects of two tuning parameters, physical pressure and isovalent substitution, are so different? In general, applying pressure reduces lattice constants, and it leads to an increase of bandwidth as well as a change in the Coulomb interactions^22^, often affecting the ground state of the system. The chemical substitution by smaller ions also leads to a decrease of lattice constants, which results in similar effect on the system as the pressure effect. Indeed, in BaFe_2_As_2_ system, the physical and chemical pressure effects on superconductivity are essentially similar^23^. To address the origin of the difference between chemical and physical pressure effects in FeSe, we determine the structure parameters of FeSe_1−*x*_S_*x*_ at room temperature by single-crystal X-ray diffraction, which are compared with the published data under pressure^24^ (Fig. 4a–c and Supplementary Table 1). As expected, both *a*-axis and *c*-axis lattice constants decrease with S content *x*, which follow the trends under physical pressure. The quantitative comparison suggests that 10% substitution corresponds to ~0.3 GPa (Fig. 4a, b). This can be compared with effects of chemical and physical pressure on the phase diagrams of BaFe_2_As_2_, where the 30% substitution of P for As^21^ and application of ~0.55 GPa^25^ both lead to the maximum ${T_{\rm{c}}}\sim30$ K.

In sharp contrast to the *a*-axis and *c*-axis lattice constants, there is a significant difference in the trends of the chalcogen height *h*_Ch_ from the iron plane (Fig. 4c). It has been pointed out in iron pnictides that the height of the anion atoms from the Fe plane plays an important role on the existence of hole-like Fermi surface around the zone corner of the unfolded Brillouin zone, which has significant influence on the nesting properties between the Fermi surfaces^26^. It has also been shown that the chalcogen height in FeSe_1−*x*_Te_*x*_ is an important factor for the magnetic interactions^27^. We find that the isovalent substitution reduces *h*_Ch_ monotonically which is opposite to the observed increasing trend due to physical pressure effect. Thus, this contrasting variation of the chalcogen height against chemical and physical pressures is likely responsible for the shift of the magnetic dome to the higher pressure side with S-substitution, because for samples with a larger S content a higher pressure is required for obtaining a large enough *h*_Ch_ to induce magnetic order. Indeed, recent theoretical calculation investigating the pressure effect in FeSe points out that the increase of *h*_Ch_ results in the appearance of Fermi surface in the Brillouin zone corner, which explains the emergence of magnetism under pressure^28^.

Another intriguing observation is that inside the SDW phase the superconducting transition becomes broad with a relatively high onset temperature (Fig. 2 and Supplementary Figs. 3–6). This is demonstrated in Supplementary Fig. 1, which shows that $T_{\rm{c}}^{{\rm{peak}}}$ defined by the peak temperature in d*ρ*(*T*)/d*T* continues to rise with pressure upon entering the SDW phase, whereas the zero resistivity $T_{\rm{c}}^{{\rm{zero}}}$ is suppressed. This implies the competing nature of the magnetic and superconducting orders, which may disturb the development of high-*T*_c_ superconductivity. Indeed, a recent detailed study of ac susceptibility measurements under pressure shows that the volume fraction in the diamagnetic shielding is suppressed in the pressure-induced SDW phase, pointing to non-bulk superconductivity inside the magnetic phase^29^.

We have shown that by combining physical and chemical pressure effects, which can change the chalcogen height in different manners, nematicity and magnetism can be tuned to control *T*_c_ in FeSe-based superconductors. The most notable feature is that the high-*T*_c_ superconductivity in the tetragonal phase emerges at the verge of both side of the SDW dome, while *T*_c_ is little influenced by the non-magnetic nematic phase. Magnetic order appears to exert two effects on superconductivity: on one hand, their competing nature suppresses the formation of bulk high-*T*_c_ superconductivity inside the magnetic phase, and on the other hand, the enhanced fluctuations near the end points of the magnetic phase may help increase *T*_c_, much more significant than the non-magnetic fluctuations near the nematic quantum critical point at $x\sim0.17$ and $P\sim0$ GPa. In view of the orthorhombicity found in the pressure-induced SDW phase, an intriguing issue that deserves further studies is whether the nematic and magnetic fluctuations cooperatively promote the superconducting pairing, as recently suggested theoretically^28^.

## Methods

### Single crystals

High-quality single crystals of FeSe_1−*x*_S_*x*_ (*x* = 0, 0.04, 0.08, 0.12 and 0.17) have been grown by the chemical vapour transport technique^18^. The *x* values are determined by the energy dispersive X-ray spectroscopy. In the crystals obtained under identical conditions, quantum oscillations have been observed in a wide range of *x*(≤0.19)^30^, indicating superior crystal quality.

### High-pressure measurements

High-pressure resistivity *ρ*(*T*, *P*) measurements have been performed under hydrostatic pressures up to 8 GPa with a constant-loading type cubic anvil apparatus which can maintain a nearly constant pressure over the whole temperature range from 300 to 2 K^9,19^. For all these high-pressure resistivity measurements, we employed glycerol as the pressure-transmitting medium, and used the conventional four-terminal method with current applied within the *ab* plane. High-pressure ac susceptibility measurements have been done by using a mutual inductance technique in a moissanite anvil cell with glycerol as the pressure-transmitting medium^23^. The pressure achieved was determined by measuring the wavelength of the *R*_1_ peak of ruby fluorescence. Synchrotron X-ray diffraction measurements under pressure have been performed at BL22XU in SPring-8 by using diamond anvil cell diffractometer equipped with a gas membrane for maintaining constant pressure on cooling^31^. Helium is used as the pressure-transmitting medium. The pressure value in the sample space is monitored by tracking the ruby fluorescence wavelength for the whole temperature range.

### Data availability

The data that support the findings of this study are available from the corresponding author upon reasonable request.

## Electronic supplementary material

Supplementary Information
