# Supplementary material for: Maximizing Tc by tuning nematicity and magnetism in FeSe1−xSx superconductors
Source: Nat Commun. 2017 Oct 26;8:1143. doi: 10.1038/s41467-017-01277-x (PMC5656606; doi:10.1038/s41467-017-01277-x)
Supplement: Supplementary file 1 — Supplementary Information [file 41467_2017_1277_MOESM1_ESM.pdf]

Supplementary Note 1: Phase diagrams

Here we show the three-dimensional temperature-pressure-substitution (T-P-x) phase diagram of Fig. 1 in two sets of two-dimensional slices; constant-x (Supplementary Fig. 1) and constant-P phase diagrams (Supplementary Fig. 2). In both cases, it is clearly seen that T_c^{zero} determined by the zero resistivity is suppressed inside the SDW phase below T_m and the suppression of magnetism is accompanied by the enhancement of T_c^{zero}. Inside the magnetic phase T_c^{peak} determined by the peak in $d\rho(T)/dT$ deviates from the zero-resistivity T_c^{zero} (Supplementary Fig. 1), likely associated with the competition between the magnetic order and superconductivity.

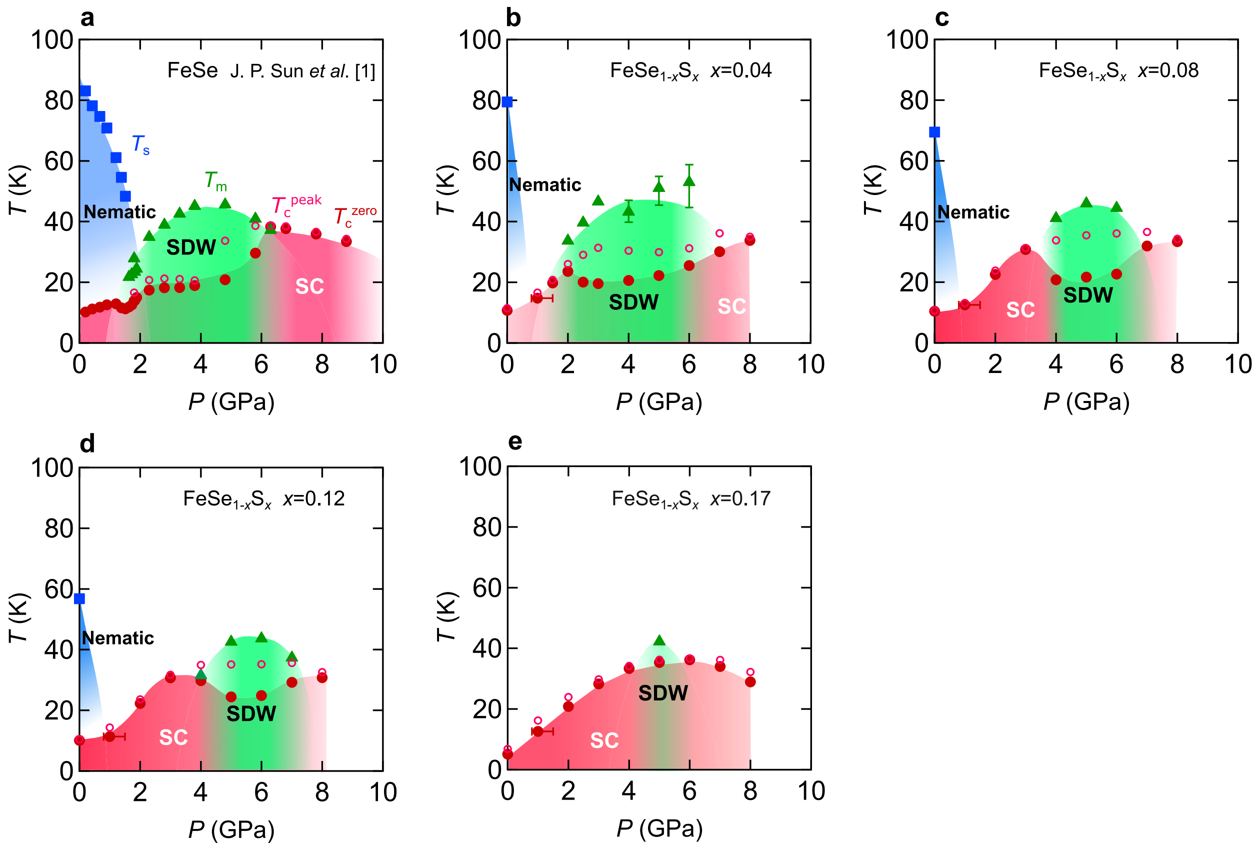

Supplementary Figure 1. Temperature versus pressure phase diagrams for constant x values in $FeSe_{1-x}S_x$. a-e, Pressure dependence of nematic (blue squares), SDW (green triangles), and superconducting transition temperatures determined by the zero resistivity (red closed circles) and by the peak in $d\rho(T)/dT$ (red open circles) at $x = 0$ (from Ref. [1]) (**a**), 0.04 (**b**), 0.08 (**c**), 0.12 (**d**), and 0.17 (**e**). The errors of T_m are estimated from the broadness of the kink anomaly in $\rho(T)$. The colour shades are the guides for the eyes.

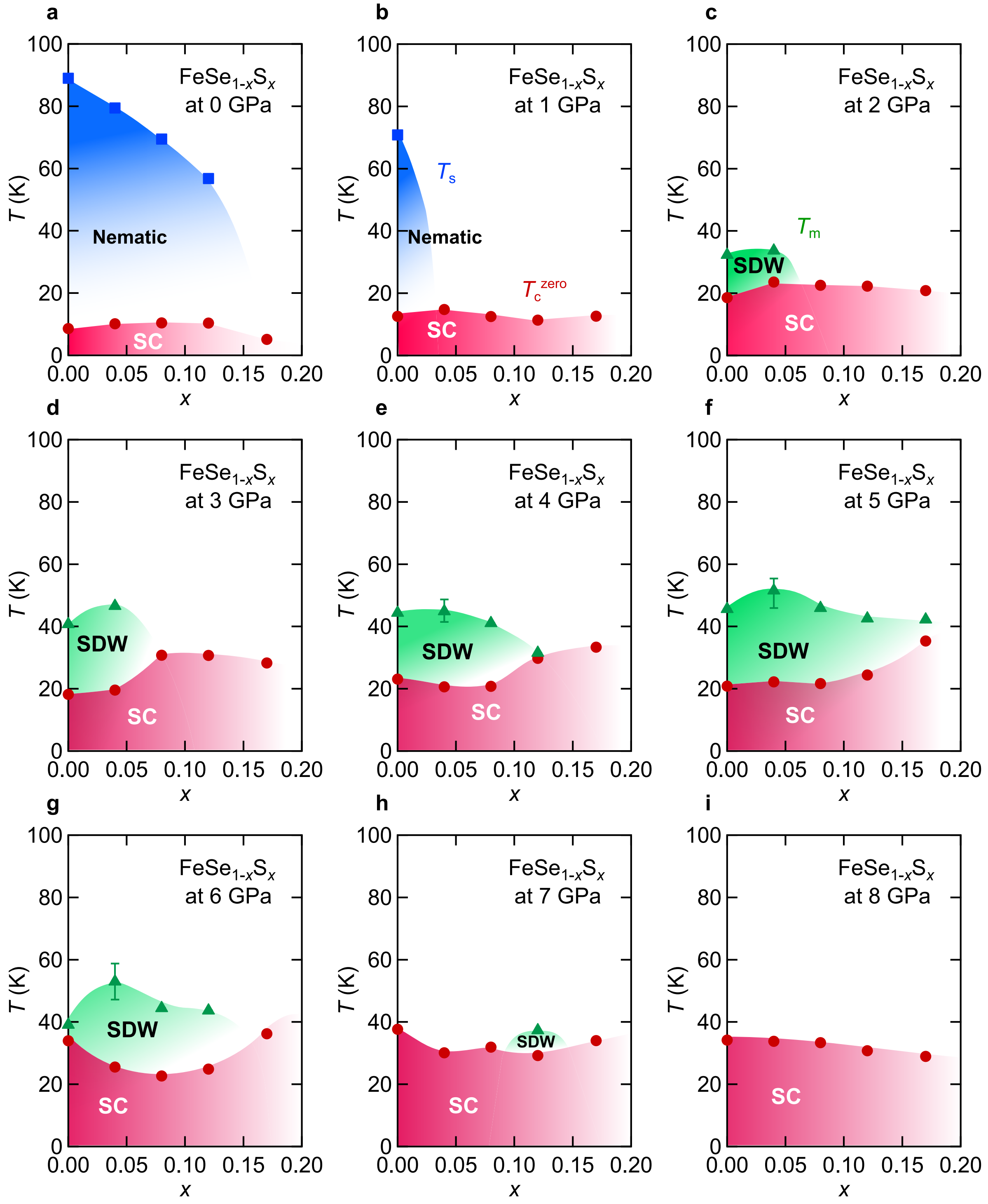

Supplementary Figure 2. Temperature versus S-content phase diagrams for constant pressure values in $FeSe_{1-x}S_x$. **a-i,** x-dependence of nematic (blue squares), SDW (green triangles), and superconducting transition temperatures determined by the zero resistivity (red circles) at $P = 0$ (**a**), 1.0 (**b**), 2.0 (**c**), 3.0 (**d**), 4.0 (**e**), 5.0 (**f**), 6.0 (**g**), 7.0 (**h**), and 8.0 GPa (**i**). The colour shades are the guides for the eyes.

Supplementary Note 2: Determination of transition temperatures

The transition temperatures are determined by the resistive anomalies under pressure. We follow the procedure reported by Sun *et al.* for FeSe [1]. An extensive set of data for the temperature dependence of resistivity ρ and its temperature derivative $\mathrm{d}\rho/\mathrm{d}T$ at different pressures are shown in Supplementary Figs. 3-6 for $x = 0.04$, 0.08, 0.12, and 0.17, respectively. The nematic (T_s) and magnetic transition temperatures (T_m) are determined by the anomalies (dip or peak) in $\mathrm{d}\rho(T)/\mathrm{d}T$. As for the superconducting transition temperature (T_c), we mainly use the zero-temperature criterion, $T_\mathrm{c}^\mathrm{zero}$, because this marks the coherent state of superconductivity and usually corresponds to the onset of the diamagnetic response.

Outside the magnetic phase, the superconducting transition is relatively sharp, indicating a good pressure homogeneity for the entire pressure range. Inside the SDW phase, however, the superconducting transition in $\rho(T)$ curves becomes very broad and has a long tail. Therefore, we also determine $T_\mathrm{c}^\mathrm{peak}$ as a peak position in $\mathrm{d}\rho(T)/\mathrm{d}T$, which is associated with the rapid decrease in ρ, and plot the pressure dependence of $T_\mathrm{c}^\mathrm{peak}$ in the phase diagrams in Supplementary Fig. 1. The large discrepancies between $T_\mathrm{c}^\mathrm{peak}$ and the zero-resistivity $T_\mathrm{c}^\mathrm{zero}$ found only inside the SDW phase are consistent with the competition between magnetic and superconducting orders that can lead to some sort of phase separation, which may also be related to the fact that the magnetic transition is likely to be first-order.

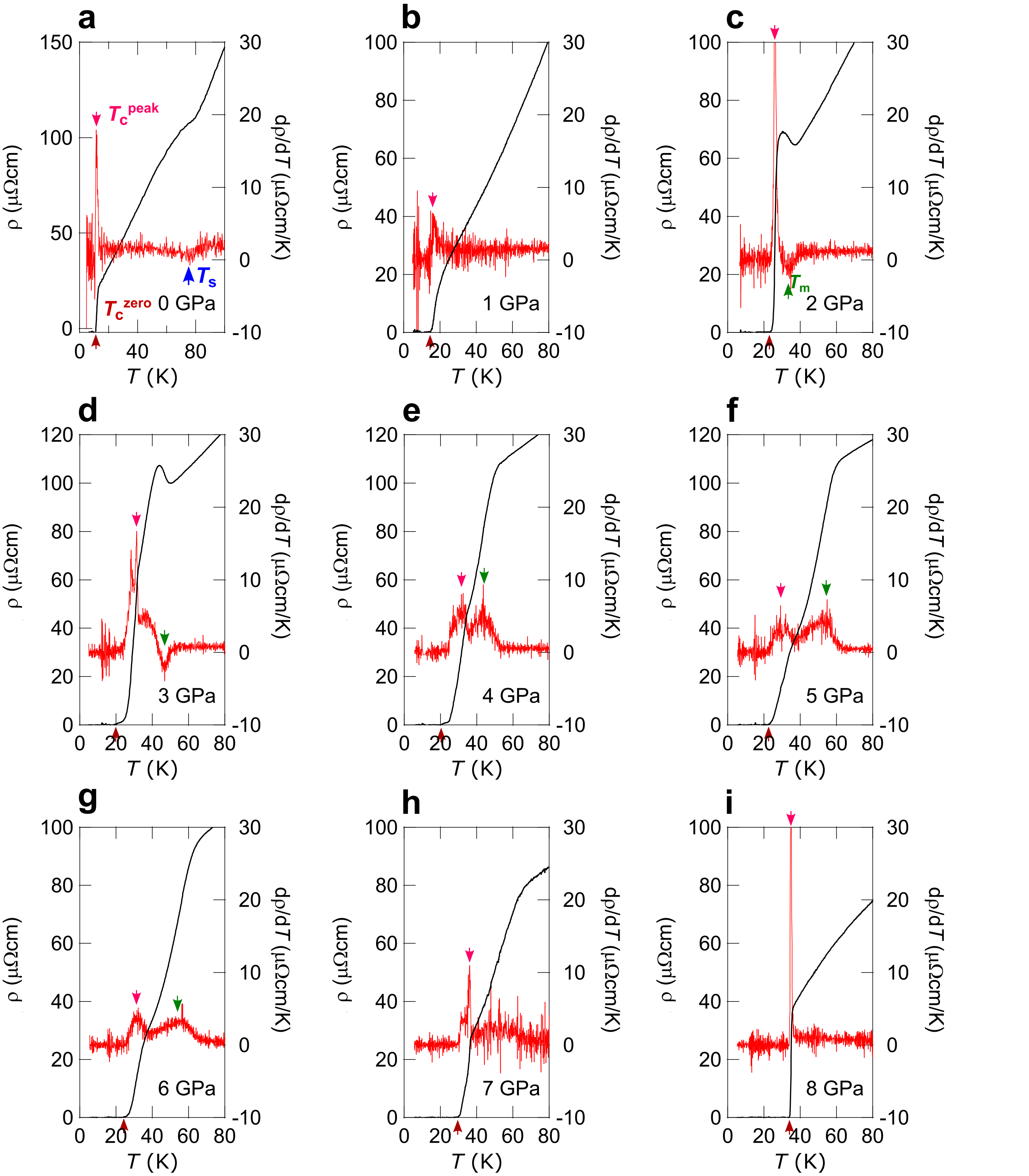

Supplementary Figure 3. Determination of transition temperatures from resistive anomalies for $x = 0.04$. Temperature dependence of resistivity (black, left axis) and $\mathrm{d}\rho/\mathrm{d}T$ (red, right axis) at $P = 0\,\mathrm{GPa}$ (**a**), 1.0 (**b**), 2.0 (**c**), 3.0 (**d**), 4.0 (**e**), 5.0 (**f**), 6.0 (**g**), 7.0 (**h**), and 8.0 GPa (**i**). Positions of T_s (blue arrow), T_m (green arrow), $T_\mathrm{c}^\mathrm{zero}$ (red arrow), and $T_\mathrm{c}^\mathrm{peak}$ (pink arrow) are indicated.

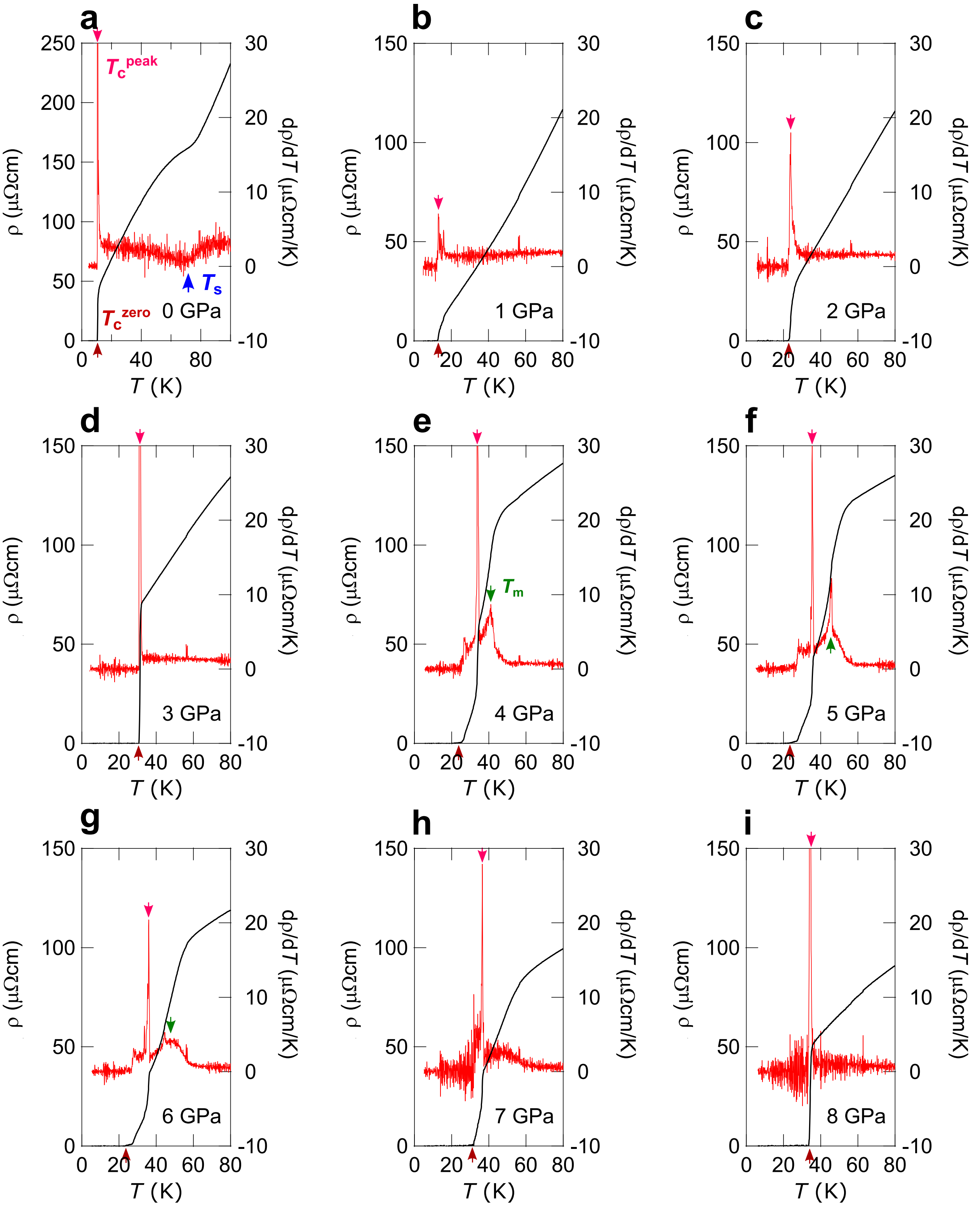

Supplementary Figure 4. Determination of transition temperatures from resistive anomalies for $x = 0.08$. Temperature dependence of resistivity (black, left axis) and dρ/dT (red, right axis) at $P = 0\,$GPa (**a**), 1.0 (**b**), 2.0 (**c**), 3.0 (**d**), 4.0 (**e**), 5.0 (**f**), 6.0 (**g**), 7.0 (**h**), and 8.0 GPa (**i**). Positions of T_{s} (blue arrow), T_{m} (green arrow), $T_{\mathrm{c}}^{\mathrm{zero}}$ (red arrow), and $T_{\mathrm{c}}^{\mathrm{peak}}$ (pink arrow) are indicated.

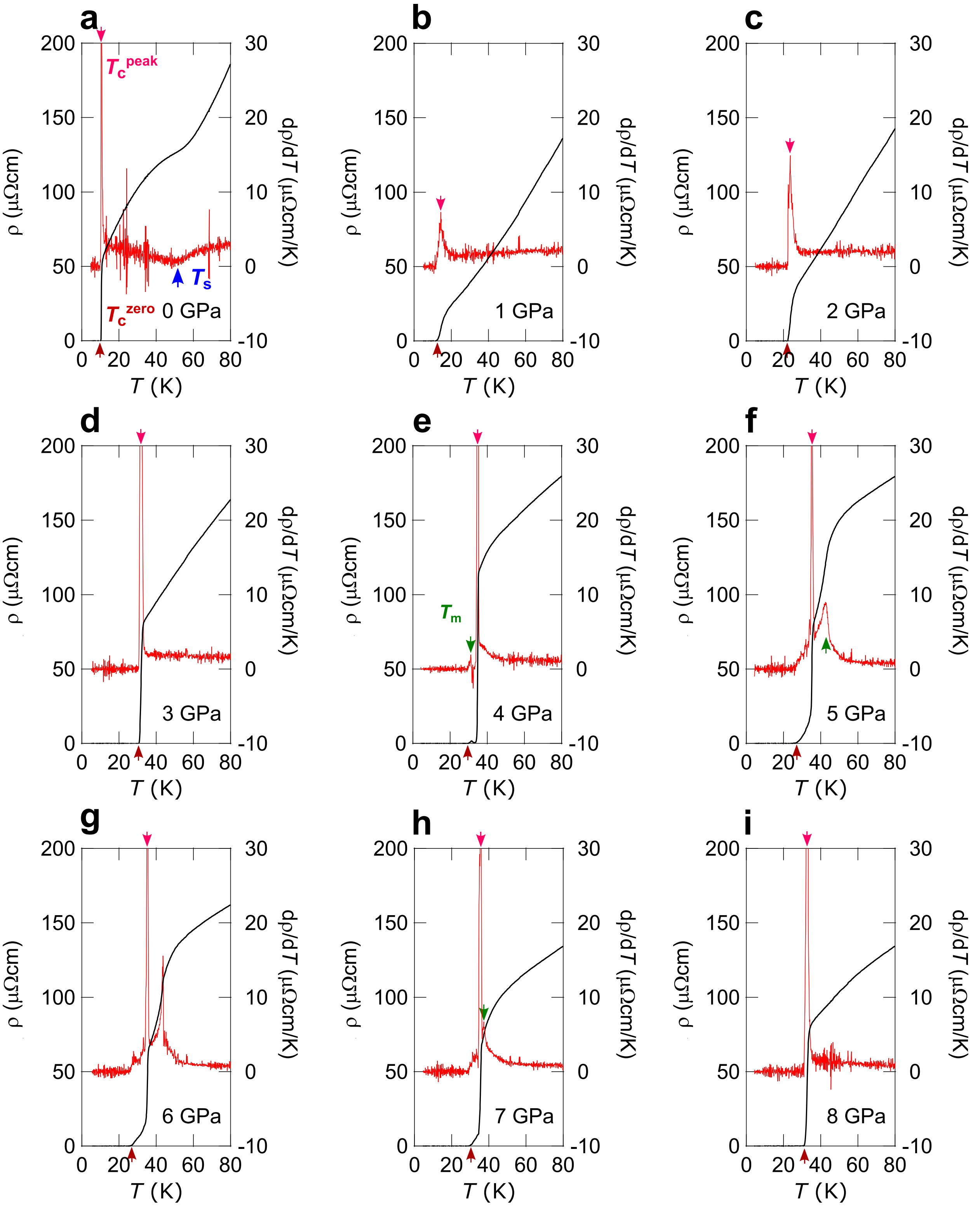

Supplementary Figure 5. Determination of transition temperatures from resistive anomalies for $x = 0.12$. Temperature dependence of resistivity (black, left axis) and dρ/dT (red, right axis) at $P = 0$ GPa (**a**), 1.0 (**b**), 2.0 (**c**), 3.0 (**d**), 4.0 (**e**), 5.0 (**f**), 6.0 (**g**), 7.0 (**h**), and 8.0 GPa (**i**). Positions of T_s (blue arrow), T_m (green arrow), T_c^{zero} (red arrow), and T_c^{peak} (pink arrow) are indicated.

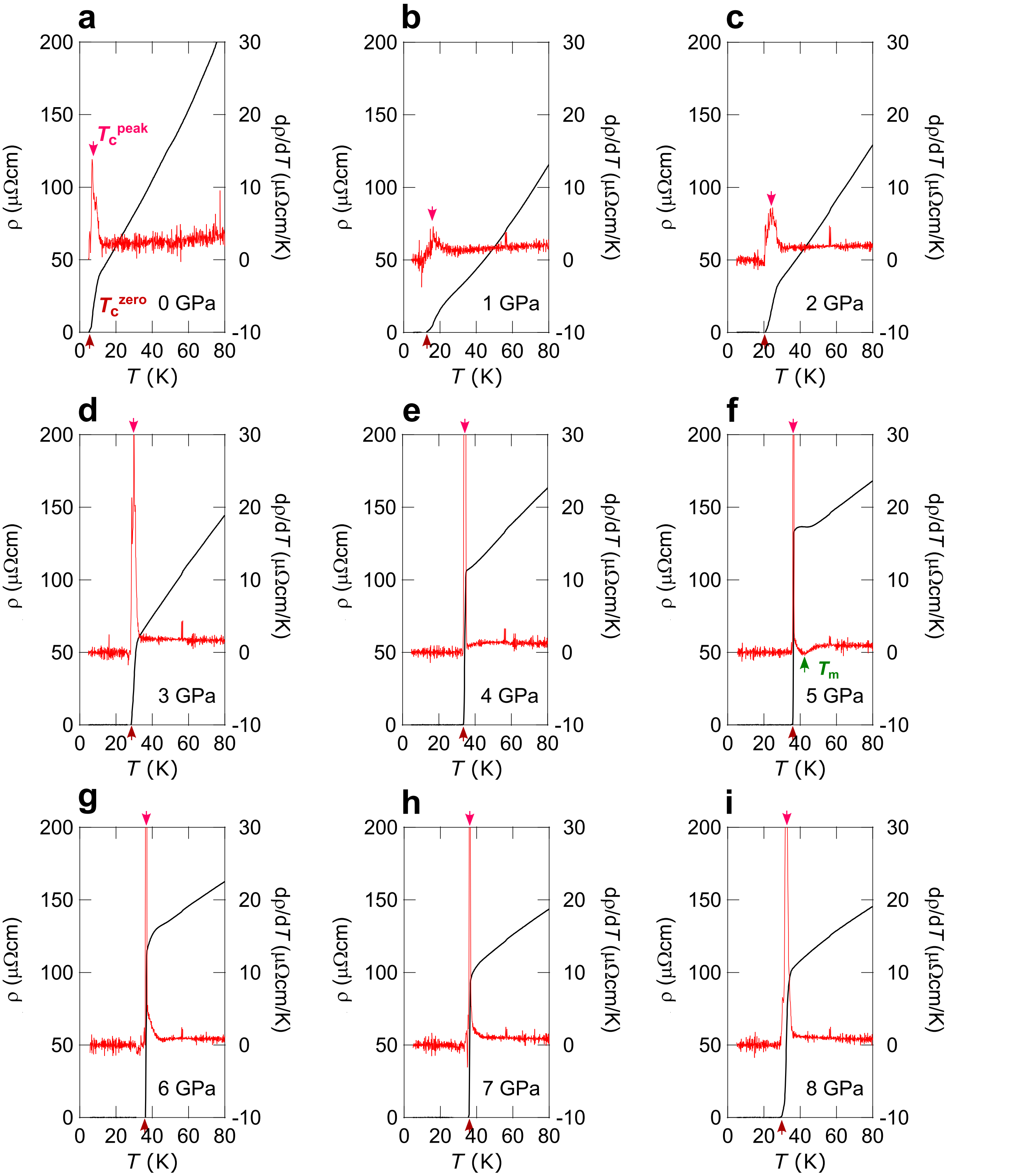

Supplementary Figure 6. Determination of transition temperatures from resistive anomalies for x = 0.17. Temperature dependence of resistivity (black, left axis) and $d\rho/dT$ (red, right axis) at $P = 0\,\mathrm{GPa}$ (**a**), 1.0 (**b**), 2.0 (**c**), 3.0 (**d**), 4.0 (**e**), 5.0 (**f**), 6.0 (**g**), 7.0 (**h**), and 8.0 GPa (**i**). Positions of T_s (blue arrow), T_m (green arrow), T_c^{zero} (red arrow), and T_c^{peak} (pink arrow) are indicated.

Supplementary Note 3: ac susceptibility measurements under pressure

The superconducting transition at high pressure is also checked by the ac susceptibility measurements for $x = 0.12$. The sample is placed in a microcoil inside a self-clamped moissanite anvil cell with glycerol as the pressure medium, and the temperature dependence of the real part of ac susceptibility $\chi_{ac}(T)$ is measured in several runs up to ~ 4 GPa. The temperature sweeps for the 1st run are shown in Supplementary Fig. 7. Clear diamagnetic signals due to superconducting transitions are observed below the pressure-dependent T_c. We note that the magnitude of diamagnetic signals at low temperatures does not show strong pressure dependence up to ~ 3 GPa, suggesting that bulk superconducting property persists in this pressure range, where T_c is enhanced significantly from the ambient pressure value. At higher pressures where the magnetic order sets in, the diamagnetic signals become significantly weaker, consistent with the competing nature of magnetic and superconducting orders, details of which are reported in a separate publication [2].

The pressure dependence of T_c determined by the ac susceptibility method is compared

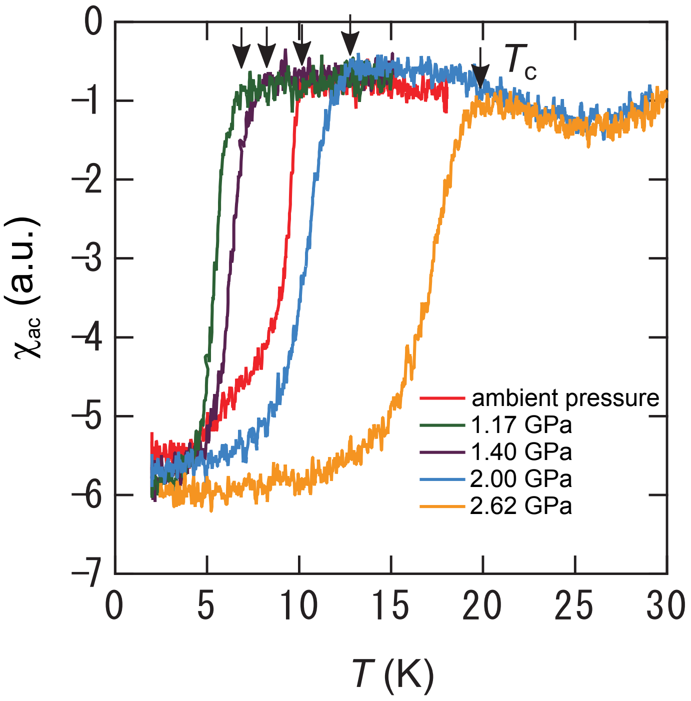

Supplementary Figure 7. Temperature dependence of ac susceptibility under pressure in $FeSe_{1-x}S_x$ for $x = 0.12$. The measurements are performed in the increasing order of pressure. The arrows indicate T_c, which is determined by the onset of the diamagnetic signal.

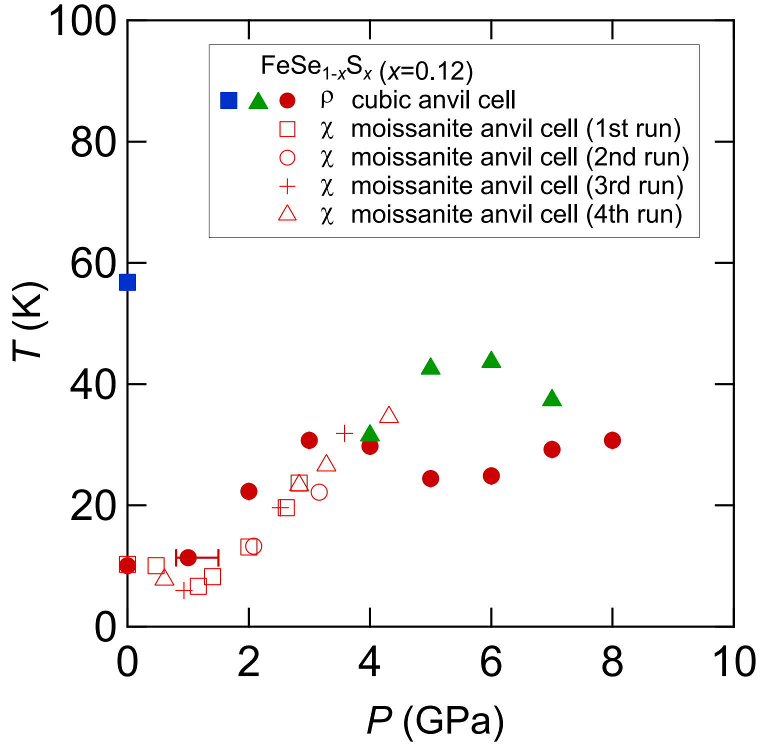

Supplementary Figure 8. Pressure dependence of T_c determined by the ac susceptibility compared with the resistivity-determined phase diagram of $x = 0.12$. T_c determined by the ac susceptibility measurements for several different runs (open symbols) are plotted as a function of pressure estimated at room temperature. For comparison the nematic T_s (blue squares), magnetic T_m(green triangles) and superconducting transition temperatures T_c^{zero} (red closed squares) determined by resistivity are also shown (from Fig. 1). The likely source of the difference between the pressure values from two different cells is discussed in the text.

with the resistivity-determined phase diagram for $x = 0.12$ in Supplementary Fig. 8. The pressure dependence of the diamagnetic T_c for several runs shows a similar trend with that of the zero-resistivity temperature T_c^{zero}. We note that the pressure for the ac susceptibility measurements is estimated by ruby fluorescence spectrum at room temperature, and thus it is likely that at low temperatures the actual pressure is lower than this estimate. Furthermore, at low pressures it is trickier to control the pressure of the cubic anvil cell. Considering the difference of pressure techniques between self-clamped type moissanite anvil cell for ac susceptibility measurements and the constant-loading type cubic anvil cell for resistivity measurements, the $T_c(P)$ results from these two different measurements are in fairly good agreement. Most importantly, the observed trend of $T_c(P)$ from two techniques is genuine.

From these results we conclude that a new, bulk high-T_c superconducting phase exists near the lower boundary of the pressure-induced magnetic dome.

Owing to the capability of finer tuning of pressure for the clamp-type cell, we find a minimum at $P \sim 1\,\mathrm{GPa}$ in the susceptibility-determined $T_c(P)$ curve. This nonmonotonic P-dependence of T_c resembles that for pure FeSe in the low-pressure range, where the nematic transition is fading. We note that very recently a low-pressure resistivity study [3] reported the phase diagrams for $x \leq 0.12$ with minima of $T_c(P)$ around 0.7-1.0 GPa, consistent with our susceptibility-determined phase diagram.

Supplementary Note 4: Lattice parameters

For $x = 0.08$, the Bragg peaks measured by synchrotron X-ray diffraction show contrasting behaviours between 3.0 GPa where high-T_c superconductivity appears and 4.9 GPa where pressure-induced magnetism is evidenced (Fig. 3). In Supplementary Fig. 9, we compare quantitatively the temperature dependence of (220) Bragg peak width between these two pressures. At high temperatures, both data show similar peak width with no splitting, but at low temperatures the 4.9-GPa data shows a clear splitting. The orthorhombicity $\delta = (a_O - b_O)/(a_O + b_O)$ rapidly develops below $T_s \approx 41\,\mathrm{K}$, implying the first-order nature of the tetragonal-to-orthorhombic transition, which is similar to the case for pressure-induced SDW phase of FeSe [4]. Except near the transition, the widths of the split peaks in the orthorhombic phase (triangles in Supplementary Fig. 9b) are both comparable to that of

Supplementary Table 1. Lattice parameters of $\mathbf{FeSe_{1-x}S_x}$. Lattice constants a, c, and the chalcogen height h_{Ch} as a function of S-content x at ambient pressure (**a**), together with the data under pressure for FeSe polycrystals taken from Millican *et al.* [5] (**b**).

**a**	S substitution				**b**	Pressure			
x	0	0.044	0.073	0.136	P (GPa)	0	0.2	0.4	0.6
a (Å)	3.7742	3.7614	3.7667	3.7624	a (Å)	3.7658	3.7610	3.7555	3.7501
c (Å)	5.5279	5.5021	5.4913	5.4675	c (Å)	5.4988	5.4794	5.4598	5.4398
h_{Ch} (Å)	1.4754	1.4702	1.4670	1.4603	h_{Ch} (Å)	1.4643	1.4668	1.4676	1.4682

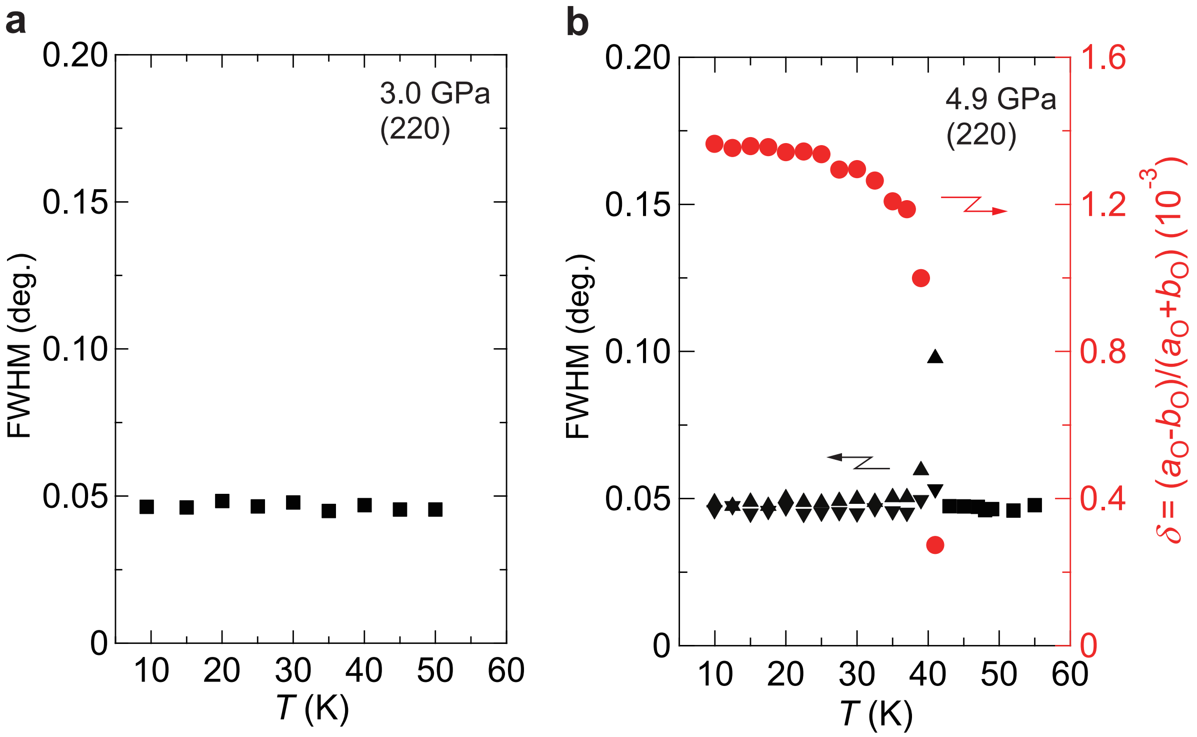

Supplementary Figure 9. Temperature dependence of Bragg-peak width and orthorhombicity in $FeSe_{1-x}S_x$ for $x = 0.08$ at high pressure. a, Full width at half maximum (FWHM) of the (220) Bragg peak as a function of temperature at 3.0 GPa. **b**, FWHM in the high-temperature tetragonal phase (black squares and triangles, left axis) and the orthorhombicity $\delta = (a_O - b_O)/(a_O + b_O)$ below $T_s \approx 41$ K determined by the splitting of the (220) Bragg peak (red circles, right axis) are plotted as a function of temperature for 4.9 GPa.

the tetragonal phase. Comparing with the results of FeSe by Kothapalli *et al.* [4], the orthorhombicity $\delta \approx 1.4 \times 10^{-3}$ at low temperatures for $x = 0.08$ at 4.9 GPa is about half of that for $x = 0$ at ambient pressure ($\delta \approx 2.7 \times 10^{-3}$) but is close to that at 3.1 GPa ($\delta \approx 1.7 \times 10^{-3}$).

The comparisons of lattice parameters between the x-dependence at ambient pressure and the P-dependence for $x = 0$ are listed in Supplementary Table 1 (see also Fig. 4). The P-dependence data are taken from results for FeSe polycrystals by Millican *et al.* [5]. The slight differences in the parameters for FeSe at ambient pressure between our single crystals and their polycrystals are possibly due to small deficiency of Se atoms in their samples.

Supplementary References

[1] Sun, J. P. *et al.* Dome-shaped magnetic order competing with high-temperature superconductivity at high pressures in FeSe. *Nat. Commun.* **7**, 12146 (2016).

[2] Yip, K. Y. *et al.* Yip, K. Y. *et al.* Weakening of the diamagnetic shielding in $FeSe_{1-x}S_x$ at high pressures. *Phys. Rev. B* **96**, 020502(R) (2017).

[3] Xiang, L. *et al.* Dome of magnetic order inside the nematic phase of sulfur-substituted FeSe under pressure. *Phys. Rev. B* **96**, 024511 (2017).

[4] Kothapalli, K. *et al.* Strong cooperative coupling of pressure-induced magnetic order and nematicity in FeSe. *Nat. Commun.* **7**, 12728 (2016).

[5] Millican, J. N., Phelan, D., Thomas, E. L., Leão, J. B., Carpenter, E. Pressure-induced effects on the structure of the FeSe superconductor. *Solid State Commun.* **149**, 707-710 (2009).
